# Moving beyond cumulative exposure scores: Profiles of adverse life experiences and associations with mental health outcomes among emerging adults

**DOI:** 10.1371/journal.pmen.0000349

**Published:** 2025-12-04

**Authors:** Cynthia M. Navarro Flores, Sara R. Berzenski

**Affiliations:** 1 Department of Psychology & Neuroscience, University of Tennessee, Knoxville, Tennessee, United States of America; 2 Department of Psychology, California State University, Northridge, California, United States of America; PLOS: Public Library of Science, UNITED KINGDOM OF GREAT BRITAIN AND NORTHERN IRELAND

## Abstract

Emerging adulthood is a period associated with increases in mental health problems, with those who have faced adverse life experiences (ALEs; adversity experienced during childhood and adulthood) being at greater risk for poor mental health outcomes. Experiencing multiple ALEs is associated with worse outcomes; however, limited research exists that looks at how patterns of ALEs relate to various mental health outcomes among emerging adults. The present study sought to understand patterns of co-occurring ALEs and their relationship to symptom severity of various mental health outcomes (e.g., depression, anxiety, substance use) by utilizing a person-centered approach (i.e., Latent Class Analysis; LCA). Data from 442 emerging adults from a university in Southern California were analyzed using Latent Class Analysis to identify various classes of ALEs. Analysis of variance with Bonferroni-adjusted post-hoc test was utilized to assess whether classes related to an array of mental health outcomes. The use of LCA suggested that a five-class mode fit the data best: (1) *Low Adversity*, (2) *Witnessing Adversity*, (3) *Experiencing Death*, (4) *Child Maltreatment and Adult Victimization*, and (5) *High Adversity.* Individuals in the *High Adversity* and *Child Maltreatment and Adult Victimization* classes had the highest average severity levels on most mental health outcomes relative to those in the other classes. These findings point to the importance of examining the specificity of adverse experiences rather than using the standard cumulative risk approach. Research implications include further assessment of specific co-morbidities of ALEs and their impact on mental health to establish consistency, as well as examining the weight of individual ALEs in predicting mental health problems.

## Introduction

Emerging adulthood is a unique stage in life filled with change, growth, and many uncertainties. Emerging adults are individuals transitioning between adolescence and adulthood, which includes ages between the late teens and their twenties [1]. This transitional stage of life is often marked by increased vulnerability to mental health problems [2]. Furthermore, emerging adults who have faced adversity during their childhood exhibit higher prevalence rates of mental health problems relative to those in national epidemiological studies [3]. Thus, examining how adversity may increase the risk for psychopathology during this crucial transitional time among emerging adults is important to inform prevention and intervention efforts. Most of the existing research examining this relationship has focused on examining adverse childhood experiences (ACEs) and using cumulative risk approaches to understand the relationship between adversity and mental health outcomes. However, this approach overlooks adversity occurring in adulthood and obscures the unique effects of specific combinations of adversities. Although recent studies have begun to use person-centered approaches to identify co-occurring adversities and their differential associations with mental health outcomes (i.e., statistical methods that identify patterns within a population or sample across a set of variables, rather than focusing on relationships between individual using a variable-centered approach) [4], these studies continue to be limited in the narrow types of adversities examined and samples lacking racial and ethnic diversity [5,6]. To address these gaps, the present study used a person-centered approach to understand patterns of co-occurring ALEs and their relationship to various mental health outcomes among a sample of emerging adults with diverse backgrounds (i.e., ethnically/racially marginalized).

### Adversity and mental health problems

A substantial body of research has documented the negative effects of exposure to adversity. Much of this literature focuses on ACEs. ACEs refer to various forms of adversity experienced during childhood, such as psychological, physical, or sexual abuse; witnessing violence against one’s mother; and living with household members who abused substances, had mental illness or suicidal behaviors, or had been incarcerated [7]. The landmark ACEs study was the first to examine how ACEs affect the well-being of adults, revealing that individuals exposed to four or more ACEs had a significantly greater risk of negative health outcomes such as alcoholism, drug use, depression, and suicide attempts [7].

Since then, a growing body of research has replicated and extended the findings of this study. For instance, a meta-analysis by Hughes and colleagues demonstrated that individuals with four or more ACEs had significantly higher odds of experiencing poor physical and mental health and substance use outcomes when compared to those with fewer ACEs [8]. Notably, several studies in this meta-analysis included additional adversities—such as child neglect, bullying, and family financial problems—which were omitted from the original ACEs study, highlighting that the original ACEs framework may omit important types of adversity. Another limitation of the ACEs framework is its exclusive emphasis on childhood adversity, without accounting for adversity that occurs in adulthood. Adversity can occur across the lifespan in many forms, each with potential impacts on mental health outcomes. The present study refers to adversity occurring during childhood and adulthood as adverse life experiences (ALEs).

Limiting the focus to ACEs may underestimate how various types of adversities and their cumulative exposure throughout the lifespan influence overall wellbeing. Indeed, a study examining the association between ACEs, ALEs, and symptoms of depression among adults found that (a) ACEs exposures increased the likelihood of experiencing more ALEs in adulthood, (b) both ACEs and ALEs were linked to depression in adulthood, and (c) ALEs partially explained the association between ACEs and depression [9]. These findings underscore the need to consider adversity beyond childhood to fully understand its impact on mental health.

Numerous studies have linked specific ALEs to various types of health and mental health problems. For example, disasters, child maltreatment, and domestic violence have been associated with depression [10–12]; disasters and child maltreatment with anxiety [10,11]; parental incarceration and divorce with antisocial behavior [13,14]; foster care placement with increased levels of externalizing behaviors [15]; and parental divorce and child maltreatment with substance use [11,13]. ALEs are also associated with a greater risk of posttraumatic stress disorder symptoms [16]. Lastly, poorer physical health has been observed when individuals experience parental death [13]. These studies highlight that the specific type of adversity is also important to consider, as these stressors have differential risk for contributing to the development of psychopathology.

Collectively, this body of research highlights the importance of considering specific types of adversity, as stressors can have differential impacts on mental health outcomes. Furthermore, prior research shows that specific ALEs often co-occur—for instance, emotional and sexual abuse commonly accompany physical abuse or domestic violence [17]. Despite this, much research relies on a cumulative or dose-response approach, focusing on assessing the total number of adversities, rather than the specific types of adversity experienced, to understand the impact of adversity on mental health outcomes. Thus, there is a need to examine multiple ALEs while accounting for the type of adversity, as this approach can provide more nuanced insights needed to inform the development of targeted prevention/intervention programs.

## Models for assessing adversity

### Cumulative risk approach

A commonly used method of examining multiple adverse experiences (i.e., ACEs, ALEs) is to combine them into a cumulative score [18]. The cumulative risk approach assesses exposure to individual ALEs by measuring if an adverse experience has occurred ( [1] *present*) or not ([0] *absent*) [18]. The total number of individual ALEs is then added, such that individuals with higher scores would be considered to be at higher risk. Utilizing this approach allows researchers to examine how many ALEs an individual has experienced, and how these levels relate to outcomes of psychopathology (e.g., high vs. low). However, this approach assumes all adversities are equally weighted in their associated risk for developing mental health problems, which may obscure important associations between specific ALEs that may most contribute to the development of psychopathology [19]. Therefore, an approach that takes into account specific combinations of ALEs may be more beneficial, as it allows researchers to assess the overall amount of adverse experiences, as well as how the specific types of ALEs relate to psychopathology [20].

### Person-centered approach

Person-centered analyses, such as a latent class analysis (LCA; for dichotomous data) or latent profile analyses (LPA; for continuous data), have been used to cluster individuals into classes accounting for the type of adversities experienced; however, these studies have mainly focused on children and ACEs exposures [21–23]. Employing a person-centered approach allows researchers to uncover combinations of adverse experiences that frequently co-occur, as well as the overall levels of adversity that individuals experience. This is important because distinct configurations of adversity have been found to have differential effects on mental health [22–24]. For example, Copeland and colleagues found six classes using an LCA in a sample of children: (1) no risk; (2) low risk with interparental problems; (3) moderate risk with single parents, poverty, and parental criminality; (4) moderate risk with uneducated parents and poverty; (5) moderate risk with parental criminality and step-parents that had psychiatric, substance use, and crime problems; and (6) high risk with poor relations and parental dysfunction [22]. Their findings suggest that youth in the moderate exposure risk groups had different mental health outcomes based on the specific types of psychosocial risk factors experienced. Those with parents with low academic attainment and who experienced poverty were more likely to report having emotional disorders and depression; youth with single parents, experiencing poverty, and parental criminality were more likely to report having ADHD; and youth of criminal justice involved parents, who lived with a step-parent with mental health problems, and/or substance use were more likely to report having depression, anxiety, and ADHD. Lanier and colleagues also used an LCA to assess combinations of ACEs and their relation to various health outcomes among youth [23]. They found that youth with higher cumulative ACEs scores were at increased risk for worse health outcomes, and that for individuals with moderate levels of ACEs, assessing the specific combinations of ACEs differentiated the health outcome experienced. For instance, youth exposed to caregiver mental health problems and poverty were at equal risk of poor health outcomes as children who experienced three or more ACEs. Hence, these studies illustrate how using a person-centered approach could be beneficial since this approach reveals configurations of ALEs that are relevant to health outcomes.

Few studies have utilized person-centered approaches to assess the co-occurrence of ACEs and their relation to child mental health outcomes [21–23,25], and even more scarce are those focusing on emerging adults [26–28]. Research employing person-centered approaches among emerging adults has identified different classes/profiles of adversity using various indicators of adversity. For example, Newcomb-Anjo and colleagues examined how co-occurring situational and childhood risk factors, as well as dispositional traits, related to mental health symptoms, wellbeing, and academic performance and stress among emerging adults using an LPA [5]. They identified four profiles: (1) low risk, (2) low social support risk, (3) financial risk, and (4) multiple risk. More recently, Tomlinson and colleagues examined classes of ACEs and their associations with depression, anxiety, somatization, and general psychological stress among a diverse sample of emerging adults (ages 18–21 years old; 61.5% minoritized individuals [6]. Using a LCA with the original ACEs [7] in addition to physical and emotional neglect, and parental separation/divorce, they identified three classes: (1) low ACEs, (2) high emotional abuse only, and (3) high multiple ACEs class.

Results from Newcomb-Anjo and colleagues and Tomlinson and colleagues illustrated that the distinct profiles/classes identified in each study have differential impacts on various outcomes of wellbeing [5,6]. For instance, Newcomb-Anjo and colleagues found that the low risk profile consistently had the most favorable outcomes, including lower depression and anxiety, higher self-esteem and life satisfaction, and lower academic stress [5]. In contrast, the multiple risk profile had the worst outcomes, including higher symptoms of depression and anxiety when compared to those with low risk and low social support risk group, and lower levels of self-esteem and life satisfaction when compared to the low risk profile. Moderate exposure risk profiles (i.e., low social support and financial risk profiles) were differently related to the outcomes measured in the study. Specifically, the low social support profile had similar outcomes as the low risk profile, whereas the financial risk profile had worse outcomes that were similar to the multiple risk profile, including higher symptoms of depression, and lower self-esteem and life satisfaction ratings. Tomlinson and colleagues also found that individuals in the high multiple ACEs class reported worse outcomes (i.e., greater somatization, anxiety, and overall psychological stress symptom severity) relative to those in the low ACEs and high emotional abuse only classes [6]. Individuals in the low ACEs and high emotional abuse only classes (i.e., the moderate exposure risk classes) reported higher symptoms of depression relative to those in the high multiple ACEs class. Lastly, individuals in the high emotional abuse only class endorsed greater anxiety and overall psychological stress severity relative to the low ACEs class. These findings highlight the increased risk that individuals experiencing higher levels of ACEs or ALEs have when compared to those with less exposure, and that those who experience moderate levels of adversity have differing outcomes depending on the adversity experienced, thus emphasizing the need to also focus on the specific types of adversity experienced.

While the studies by Newcomb-Anjo and colleagues and Tomlinson and colleagues advance our understanding of co-occurring adversity among emerging adults and their association with outcomes of wellbeing, they are constrained by the limited types of adversities assessed and the samples used for the studies [5,6]. For instance, Newcomb-Anjo and colleagues examined limited ALEs (i.e., childhood verbal abuse, childhood subjective SES, financial strain, perceived social support, and negative life events), leaving out potential ALEs included in the ACEs framework (e.g., child physical abuse) [5]. Furthermore, findings by Newcomb-Anjo and colleagues are limited as the sample was predominantly White and from Canada [5], which restricts generalizability to more diverse populations that may be at higher risk of experiencing ALEs [29,30]. While Tomlinson and colleagues included a more diverse sample (61.5% marginalized individuals), their study only measured ACEs. This may exclude important stressors occurring later in life that can contribute to mental health outcomes among adults, and could lead to capturing fewer clusters of adversities [6]. Thus, research examining a larger range of ALEs among samples with higher representation of ethnically and racially diverse emerging adults is needed.

The present study sought to identify classes of participants based on their exposure to a wide array of ALEs and assess how these classes related to various types of mental health outcomes among emerging adults. We expected that classes would form based on the amount and types of adversity experienced by the participants. Specifically, we expected that low adversity, moderate adversity, and high adversity exposure classes would emerge from the person-centered analysis. Furthermore, among those with moderate adversity exposure, we hypothesized that there would be multiple configurations of classes separated by distinct types of ALEs; however, investigating the specific types of adversity that would co-occur was exploratory as limited literature exists. When examining the relationship between the different classes and the various types of mental health outcomes, it was expected that classes with more ALEs (i.e., high and moderate exposure classes) would have higher levels of psychopathology, and that the moderate ALEs exposure classes would differentially relate to psychopathology outcomes.

## Materials and methods

### Participants

The present study included data from 442 college undergraduate students (*M*age = 19.5 years, *SD* = 2.02) who were enrolled in an introductory psychology course at a university in Southern California. To enroll in the study, the participants had to be at least 18 years old, students at the university, and currently enrolled in an introduction to psychology course. Participants were 61.8% female and 38.2% male, 60.2% Latinx, 13.6% White, 10.6% Asian/Pacific Islander, 5.7% Black, 2.7% Middle Eastern, 6.1% mixed, and 1.1% other.

### Procedures

Recruitment took place through the university’s research management system between November 22, 2016, and December 10, 2016. Students were able to see a description of the study online, where they were provided information on the purpose of the study (“to investigate how different negative life experiences, behaviors, coping styles, and personality traits affect mental health and positive adaptation”). Those who were interested in participating were scheduled for an in-person survey. Data were collected from 25 students at a time in a university computer laboratory. Participants were provided additional information about the study at this time and were assured that the survey would be completely anonymous. Participants completed informed consent via the online survey by selecting whether or not they agreed to participate in the study. The participants who consented to participate in the study then completed an 80-minute in-person online survey and one paper survey. Students were compensated with eight course credits (i.e., one credit for every 10 minutes of participation). Participants were required to stay for the full 80 minutes to minimize any incentive to rush through questionnaires. Participants were informed that they could skip any question they did not feel comfortable answering and were allowed to discontinue the study at any time by notifying the researcher administering the survey. At the end of the study, participants were debriefed and provided with the contact information for the university counseling center. They were encouraged to reach out if they experienced any distress due to the questions asked on the survey.

### Ethics statement

The protocol for the current study was approved by the California State University, Northridge – Institutional Review Board (Protocol # 1516–245). The study was conducted anonymously, and thus no names or signatures were collected from the participants at any time during the data collection. Participants instead used an anonymous identification number. Once participants understood the details of the study, they provided informed consent to participate by selecting the *yes* or *no* option on the online survey.

### Measures

#### Adverse life experiences.

The *Life Stressor Checklist-Revised* (LSC-R) [31] was administered to assess exposure to ALEs via the online survey. This questionnaire included 30 self-reported questions, each consisting of a different possible life experience. Items asked about experiences with natural disasters, physical or sexual abuse, death of family members, and other events. Participants were asked to state whether an event had occurred to them or not (*yes* or *no*). Data was coded as each ALE being (1) *present* or (0) *absent*. Information for 29 questions was collected. One question asking if the participant had ever been separated or divorced was accidentally omitted from the questionnaire, therefore, this information was not collected. Not all questions were incorporated into the model. For example, two open-ended questions asked participants to report any adverse events that they had experienced and had not yet been asked by the questionnaire, and events that occurred to other people who they were close to. Adversities with less than 5% of endorsement were also omitted from the model as it would be difficult for the model to converge. The omitted experiences were being in jail and foster care, experiencing physical neglect, having an abortion, having a handicapped child, and experiencing being separated from their child. In addition, the questions asking participants about specific life events that occurred to them before the age of 16 and after the age of 16 were combined to create one variable (e.g., sexual touch before the age of 16 and after the age of 16 were combined to create a sexual touch variable). This was done because of conceptual similarity, but also because the participants erroneously reported incidents that were not within the age range of each question. Thus, accuracy was preserved by creating single, lifespan versions of these variables. Ultimately, 18 ALEs were included in the analyses: (1) disaster, (2) seen accident, (3) accident, (4) family in jail, (5) parental divorce, (6) financial problems, (7) physical/mental illness, (8) emotional abuse/neglect, (9) caring for ill, (10) unexpected death, (11) death, (12) domestic violence, (13) seen robbery, (14) been in robbery, (15) sexual harassment, (16) physical abuse, (17) sexual abuse, and (18) sexual touch. The LSC-R has been used with various populations (e.g., women with substance use and comorbid mental health problems, adults in methadone maintenance treatment, international populations) and has demonstrated good reliability, as well as strong convergent and concurrent validity [32–34].

#### Mental health outcomes.

The *Adult Self Report (ASR)* [35] is a widely used scale to assess mental health problems in adults. This questionnaire measures symptom severity for mental health and substance use problems, including Diagnostic and Statistical Manual of Mental Disorders (DSM)-oriented scales that assess specific disorders based on DSM criteria, as well as the frequency of substance use. Participants were asked to complete a paper version of the ASR. Participants rated statements based on how much each statement described them in the past six months on a three-point scale ranging from (0) *not true* to (2) *very true or often true*. For the purpose of the present study, only the DSM-oriented scales and substance use scales were used to evaluate the participants current mental health. The DSM-oriented scales included 14 items measuring depressive problems, 7 items for anxiety problems, 9 items for somatic problems, 7 items for avoidant personality problems, 13 items for attention-deficit/hyperactivity disorder (ADHD), and 20 items for antisocial personality problems. The DSM-oriented scales have been shown to have good reliability, ranging from.68 to.84, and test-retest reliability from.78 to.94 [35]. The substance use scales each included one item asking participants to report on the number of days they used tobacco, alcohol, and drugs in the past six months. These scales have demonstrated good test-retest reliability ranging from.82 to.99. Raw scores were used for the DSM-oriented scales as recommended by the manual [36], as T-scores become truncated at the lower end of the scale and this provides greater variability. T-scores were used for the substance use scales due to significant variability in the number of substances utilized.

### Analytical strategy

Mplus Version 6.12 [37] was used to fit LCA models. For the LCA, models with lower Akaike Information Criterion (AIC), Bayesian Information Criterion (BIC), and Sample-Size Adjusted BIC values, higher entropy values that were closer to 1, and statistically significant Lo-Mendell-Rubin likelihood ratio test (LMRT) tests indicating that the model with more classes significantly improved the fit when compared to a model with one fewer class, were preferred [38]. Once the distinct classes of ALEs were obtained, Analysis of variance (ANOVA) with Bonferroni-adjusted post-hoc tests were used in SPSS version 22 [39] to compare mean level differences of mental health outcomes for the different classes of ALEs.

## Results

### Preliminary analyses

The patterns of missing data were identified by conducting a Little’s Missing Completely at Random (MCAR) test. The Little’s MCAR test, *χ*^2^ (1676) = 1845.86, *p* = .022, which indicated that the data was not missing completely at random (see Tables 1 and 2). Further evaluations were done to assess missing data patterns by conducting independent samples t-tests to see if the variables missing more than 5% of responses related to all other variables. All the variables except caregiving of handicapped family members had less than 5% missing data. There were significant relations (*p* < .05) between missing data on indicators representing care giving for a handicapped family member and experiencing physical abuse after age 16, sexual touch after age 16, and sexual abuse before and after age 16. Given that the data was not Missing at Random (MAR), maximum likelihood estimation was used to impute missing values, as this method provided a less biased method of handling missing data relative to listwise or pairwise deletion [40].

**Table 1:** Descriptive and Missing Data for ALEs.

				Missing	
ALEs	*N*	Frequency	Percent	Count	Percent
Disaster	442	85	19.23%	0	0%
Seen Accident	429	166	38.69%	13	2.9%
Been in Accident	438	89	20.32%	4	0.9%
Family in Jail	431	134	31.09%	11	2.5%
Parental Divorce	439	145	33.03%	3	0.7%
Financial Problems	438	85	19.41%	4	0.9%
Physical/Mental Illness	440	50	11.36%	2	0.5%
Emotional Abuse/Neglect	439	105	23.92%	3	0.7%
Caregiving for Handicapped	418	46	11.00%	24	5.4%
Unexpected Death	425	127	29.88%	17	3.8%
Death	427	183	42.86%	15	3.4%
Domestic Violence	441	128	29.02%	1	0.2%
Seen Robbery	437	67	15.33%	5	1.1%
Been in Robbery	436	46	10.55%	6	1.4%
Physical Abuse Before 16	440	47	10.68%	2	0.5%
Physical Abuse After 16	435	30	6.90%	7	1.6%
Sexual Harassment	437	77	17.62%	5	1.1%
Sexual Touch Before 16	438	49	11.19%	4	0.9%
Sexual Touch After 16	436	24	5.50%	6	1.4%
Sexual Abuse Before 16	437	19	4.35%	5	1.1%
Sexual Abuse After 16	433	24	5.54%	9	2.0%

*Note.* The number and percentage of missing cases.

**Table 2:** Descriptive and Missing Data for Outcome Variable.

				Range		Missing	
Mental Health and Substance Use Outcomes	*N*	Mean	*SD*	Minimum	Maximum	Count	Percent
Depressive Problems	442	6.97	5.50	0	26		0
Anxiety Problems	442	6.41	3.26	0	14	0	0
Somatic Problems	436	2.89	3.11	0	16	6	1.4
ADHD Problems	442	4.30	3.03	0	25	0	0
Avoidant Personality Problems	442	7.98	5.13	0	14	0	0
Antisocial Personality Problems	442	5.59	4.86	0	25	0	0
Tobacco Use	435	50.37	1.28	50	59	7	1.6
Alcohol Use	437	54.74	6.33	50	85	5	1.1
Drug Use	433	55.84	11.18	50	100	9	2.0

*Note.* The number and percentage of missing cases.

Mental health and substance use outcomes were measured using the Adult Self Report measure.

Tobacco, alcohol, and drug use variables are reported as T-scores. All other variables are presented as raw scores.

### Latent class analysis of ALEs

The LCA model fit was evaluated for possible two to seven-class models (fit statistics for each model are shown in Table 3). A five-class solution was selected as it was the best-fitting model overall. While the entropy of the five-class model was not the highest out of all the models (the seven-class model had the highest entropy), the five-class model had lower BIC and Sample-Size Adjusted BIC values relative to the seven-class model. As the number of classes increased, the AIC values continued to decrease, and the BIC values increased. The Sample-Size Adjusted BIC value decreased as more classes were added; however, this metric began to increase once the sixth class was added to the model. In addition, the LMRT values were nonsignificant except for the five-class model, indicating that the five-class model was a significantly better fit than the four-class model. Therefore, the decision to select the five-class model was made, as this model was parsimonious, had adequate entropy, had a significant LMRT value, lower AIC values than models with fewer classes, and the lowest Sample-Size adjusted BIC value.

**Table 3:** Fit Statistics for Latent Class Models.

Classes	AIC	BIC	Sample-Size Adjusted BIC	LMRT *p* value	Entropy
2	7384.78	7536.16	7536.16	<.001	0.773
3	7354.91	7584.03	7406.31	.505	0.659
4	7332.36	7639.21	7401.19	.483	0.730
**5**	**7312.95**	**7697.53**	**7399.22**	**.041**	**0.751**
6	7301.53	7763.85	7405.24	.330	0.754
7	7293.46	7833.51	7414.61	.718	0.780

*Note.* AIC = Akaike Information Criterion; BIC = Bayesian Information

Criterion; LMRT = Lo-Mendell-Rubin likelihood ratio test; LCA = Latent Class Analysis.

Row highlighted in bold was selected as the best-fitting model.

Item response probabilities (see Table 4) indicated that Class 1 was classified with low probabilities on every ALEs indicator; thus, this class was identified as *Low Adversity*.

**Table 4:** Item-Response Probabilities for 5 Class Model.

ALEs	Class 1 Low Adversity (51.36%)	Class 2 Witnessing Adversity (4.30%)	Class 3 Experiencing Death (27.37%)	Class 4 Child Maltreatment and Adult Victimization (12.90%)	Class 5 High Adversity (4.07%)
Disaster	.07	**.57**	.23	.28	**.55**
Seen Accident	.25	**1.00**	.41	.45	**.87**
Accident	.11	.38	.26	.16	**.71**
Family in Jail	.19	**.51**	.41	.28	**.79**
Parental Divorce	.22	**.52**	.34	**.55**	**.50**
Financial Problems	.02	.00	.37	.31	**.61**
Physical/ Mental Illness	.03	.05	.14	.17	**.69**
Emotional Abuse/Neglect	.04	.00	.33	**.63**	**.75**
Caring for Ill	.03	.00	.15	.15	**.63**
Unexpected Death	.18	.05	.45	.34	**.69**
Death	.31	.36	**.63**	.29	**.80**
Domestic Violence	.12	.20	.39	**.54**	**.76**
Seen Robbery	.10	**.76**	.09	.16	**.54**
Robbery	.06	.42	.07	.10	.48
Sexual Harassment	.04	.00	.15	**.64**	**.56**
Physical Abuse	.04	.00	.11	.47	**.51**
Sexual Abuse	.01	.09	.00	.40	.37
Sexual Touch	.04	.00	.00	**.64**	**.55**

*Note.* Items in bold indicate high probabilities of experiencing that type of adversity.

Class 2 was characterized by high probabilities of experiencing a disaster, seeing an accident, seeing a robbery, and moderate probabilities of having a family member in jail and experiencing parental divorce. Therefore, this class was identified as *Witnessing Adversity*, as the majority of ALEs that occurred were witnessed rather than experienced. Class 3 was categorized by moderately high probabilities of experiencing the death of an individual who was close to the participant; thus this class was labeled *Experiencing Death*. Class 4 was characterized by high probabilities of parental divorce, emotional abuse or neglect, domestic violence, sexual harassment, and sexual touch, and moderate probabilities of physical abuse. This class was classified as *Child Maltreatment and Adult Victimization*, as many of the ALEs endorsed are considered a form of abuse or victimization, and these events took place during their childhood or later as an adult. Lastly, Class 5 was classified by high probabilities on almost all ALEs, with the exception of moderate probabilities of parental divorce, being robbed, and physical abuse, and a low probability of sexual abuse exposure. Consequently, this class was identified as *High Adversity*, as participants experienced the majority of the life adversities.

### Relationship between the ALEs classes and mental health outcomes

The second aim of this study was to understand the relationship between the classes of ALEs uncovered through the person-centered approach and associated symptom severity among various types of mental health concerns. The results suggested there were significant differences in symptom severity between the classes on all psychopathology and substance use outcomes (Table 5). Across all types of psychopathology, the *High Adversity* and *Child Maltreatment and Adult Victimization* classes had the highest levels of mental health concerns and did not significantly differ from one another on any outcome. The *Witnessing Adversity* and *Low Adversity* classes consistently had the lowest endorsement of psychopathology. The *Witnessing Adversity* class had significantly lower levels of psychopathology relative to the *Child Maltreatment and Adult Victimization* class on every outcome except antisocial personality problems and tobacco/drug/alcohol use outcomes, and lower levels of pathology than the *High Adversity* class on every outcome except depressive symptoms, avoidant problems, and tobacco/drug/alcohol use. The *Low Adversity* class had lower levels of psychopathology relative to the *Child Maltreatment and Adult Victimization* class on every outcome except tobacco, and lower levels of pathology than the *High Adversity* class on every outcome except tobacco and drug use. The *Low Adversity* class also had lower levels of depressive symptoms and anxiety problems relative to the *Experiencing Death* class. The *Experiencing Death* class had moderate levels of pathology across outcomes. Specifically, the *Experiencing Death* class had lower levels of depressive symptoms, somatic problems, and antisocial problems relative to the *Child Maltreatment and Adult Victimization* class, and lower levels of anxiety, somatic problems, and antisocial problems than the *High Adversity* class.

**Table 5:** Means, Standard Deviations, and Analysis of Variance (ANOVA) Results on Various Types of Psychopathologies by Adverse Life Experiences Class.

	1. Low Adversity		2. Witnessing Adversity		3. Experiencing Death		4. Child Maltreatment and Adult Victimization		5. High Adversity		*F*
Mental Health and Substance Use Outcomes	*M*	*SD*	*M*	*SD*	*M*	*SD*	*M*	*SD*	*M*	*SD*	
Depressive Problems	5.52^**D**,**H,C**^	5.50	6.37^**C**^	6.01	7.52^**C,L**^	5.20	10.42^**W,D,L**^	6.74	11.17^**L**^	5.64	14.01^***^
Anxiety Problems	5.62^**D**,**H,C**^	3.02	5.32^**H,C**^	3.90	6.88^**H,L**^	3.21	8.07^**W,L**^	2.96	9.17^**W,D,L**^	2.99	12.55^***^
Somatic Problems	2.11^**H,C**^	2.60	2.53^**H,C**^	2.59	3.02^**H,C**^	2.72	5.04^**W,D,L**^	3.94	5.50^**W,D,L**^	4.46	15.09^***^
Avoidant Personality Problems	3.74^**H,C**^	2.80	3.16^**C**^	2.59	4.58	3.09	5.86^**W,L**^	3.33	5.83^**L**^	2.41	8.36^**^
ADHD Problems	6.92^**H,C**^	4.71	6.53^**H,C**^	4.51	8.45	4.89	10.56^**W,L**^	5.97	11.61^**W,L**^	5.03	9.59^***^
Antisocial Personality Problems	4.79^**H,C**^	4.25	5.42^**H**^	5.27	5.38^**H,C**^	4.50	7.98^**D,L**^	5.68	9.78^**D,L**^	6.90	9.01^***^
Tobacco Use	50.26	1.12	51.05	1.96	50.39	1.35	50.29	1.12	51.00	1.90	2.88^*^
Alcohol Use	53.66^**H,C**^	5.34	54.11	5.14	55.12	6.81	57.31^**L**^	7.30	58.76^**L**^	8.79	6.06^***^
Drug Use	53.86^**C**^	8.60	59.95	15.87	57.05	12.06	59.13^**L**^	13.24	59.07	17.49	4.37^**^

*Note.* ^*^ *p* < .05., ^**^ *p* < .01, ^***^ *p* < .001.

^**W**^ = significant Bonferroni adjusted post-hoc difference at *p* < .05 when compared to witnessing adversity class.

^**D **^= significant Bonferroni adjusted post-hoc difference at *p* < .05 when compared to experiencing death class.

^**H** ^= significant Bonferroni adjusted post-hoc difference at *p* < .05 when compared to high adversity class.

^**C**^ = significant Bonferroni adjusted post-hoc difference at *p* < .05 when compared to child maltreatment and adult victimization class.

^**L**^ = significant Bonferroni adjusted post-hoc difference at *p* < .05 when compared to low adversity class.

Mental health and substance use outcomes were measured using the Adult Self Report measure.

Tobacco, alcohol, and drug use variables are reported as T-scores. All other outcome variables are presented as raw scores.

## Discussion

The current study investigated how the co-occurrence of various ALEs relates to the severity of various mental health outcomes, utilizing a person-centered approach within a sample of emerging adults. Utilizing a person-centered approach allowed for classes of ALEs to be uncovered, capturing clusters of specific ALEs that commonly co-occur that would have been obscured by a cumulative risk approach. The use of LCA suggested that a five-class model fit the data best: (1) *Low Adversity*, (2) *Witnessing Adversity*, (3) *Experiencing Death*, (4) *Child Maltreatment and Adult Victimization*, and (5) *High Adversity.* Individual membership in these classes was differentially associated with mental health outcomes. Specifically, individuals in the *High Adversity* and *Child Maltreatment and Adult Victimization* classes had the highest average severity on most psychopathology outcomes relative to those in the other classes. These findings further support the utility of person-centered analyses in improving our understanding of how different combinations of ALEs relate to an array of psychopathologies.

### Configurations of adversity

Consistent with the study hypotheses, ALEs were represented by more than four-classes, with the best-fitting model being the five-class model. In this model, a *Low Adversity* and *High Adversity* class emerged as hypothesized. Three other classes emerged from the model: *Witnessing Adversity*, *Experiencing Death*, and *Child Maltreatment and Adult Victimization*. These classes experienced fewer ALEs than the *High Adversity* class and more adverse experiences than the *Low Adversity* class, thus these classes were all considered as experiencing a moderate number of ALEs exposures. However, whereas these classes may be in the lower range on the number of life adversities that they experienced, some of the adverse events could be severe and traumatizing, particularly in the case of the *Child Maltreatment and Adult Victimization* class. This finding illustrates the importance of not only measuring the quantity of adversity exposures but also the need to qualitatively assess the types of ALEs that individuals encounter.

Having found *High Adversity* and *Low Adversity* classes in this model is consistent with the Copeland and colleagues [22] study, which found six classes, two of which were a high and a low risk class. This is also consistent with the *high* and *low adversity* classes identified by Tomlinson and colleagues [6]. Further, it is not surprising that a *Child Maltreatment and Adult Victimization* class emerged from the analysis as subtypes of child maltreatment often co-occur [41], and having experienced child abuse increases the likelihood of subsequent victimization as an adult [42]. While the *Witnessing Adversity* class represents a small proportion of the sample, roughly 4%, this combination of adversities may be unique as the individuals did not have direct exposure to most ALEs, but witnessed adversity occurring to others in their environment. This class may represent community violence or neighborhood risk. Exposure to community violence is known to affect family functioning [43], which may help explain why individuals in this class also had an increased likelihood of having family members in jail and having experienced parental divorce. Lastly, our uncovering of the *Experiencing Death* class suggests that this adversity is distinct from other exposure patterns of ALEs examined.

### Differences in levels of mental health concerns for the distinct ALEs classes

Associations between ALE class membership and psychopathology were found, such that classes that were considered to have experienced higher levels of adversity also had higher levels of psychopathology across various types of mental health problems. The *High Adversity* and the *Child Maltreatment and Adult Victimization* classes consistently exhibited higher levels of mental health problems than the *Low Adversity* class, and in some cases, more than the two other moderate adversity exposure classes. More importantly, the *Child Maltreatment and Adult Victimization* class was similar to the *High Adversity* class across most outcomes, and the *Witnessing Adversity* class was similar to the *Low Adversity* class across most outcomes. These findings are consistent with previous literature suggesting that individuals have worse mental health outcomes when exposed to multiple ALEs [18], and that high-risk individuals have elevated levels of psychopathology when compared to lower-risk classes [22,23].

Our findings suggesting that the *Child Maltreatment and Adult Victimization* class also had elevated levels of psychopathology were not unexpected, as child maltreatment is associated with increased internalizing and externalizing behavior severity [44]. Furthermore, these findings highlight that assessing the specific ALEs rather than examining a cumulative score, that does not account for the type of ALEs, may provide researchers with greater information about the weight that each ALE carries. Most research looking at adversity have relied on cumulative risk and findings consistently show that experiencing greater number of ALEs is associated with increased symptoms of psychopathology [8,18]. However, findings from our study demonstrate that it is not only the number of ALEs that one is exposed to, but also the specific types of ALEs experienced that can help explain differences in mental health outcomes.

This study shows that specific ALEs, such as victimization, may carry more weight, as their impact seems to be just as detrimental for mental health outcomes as experiencing high levels of general adversity. Furthermore, while not statistically significant, there was a trend between the *Witnessing Adversity* class having a lower severity of psychopathology when compared to the *Experiencing Death* class. This may be due to the small sample for the *Witnessing Adversity* class, thus future research should assess this pattern of exposure with a larger at-risk sample. It is also possible that witnessing adversity does not have the same impact on mental health outcomes relative to direct exposure to ALEs.

Similar trends of findings have been observed in prior research by Newcomb-Anjo and colleagues [5], where individuals in the moderate exposure risk profiles (i.e., *low social support* and *financial risk* profiles) had vastly different outcomes. Specifically, adults in the financial risk profile showed similarly poor outcomes to the class experiencing the most adversity (i.e., multiple risk profile). Similarly, in the present study, some of the moderate exposure risk classes (i.e., *Child Maltreatment and Adult Victimization*) demonstrated comparable levels of mental health problems to the class experiencing the most adversity (i.e., *High Adveristy class*), while the *Experiencing Death* class showed somewhat higher symptom levels than the other moderate exposure risk class (i.e., *Witnessing Adversity*). Furthermore, the *Witnessing Adversity* class had similar outcomes to the *Low Adversity* class, despite having greater exposure to ALEs than the *Low Adversity* class. These findings suggest that mental health outcomes are influenced not only by the total number of adversities experienced, documented in research supporting a dose-response relationship [8], but also by the specific types of adversities, as certain experiences can disproportionately impact mental health regardless of overall adversity exposure.

## Limitations and future directions

Although the findings from this study have significant implications, it is necessary for future research to confirm and expand upon our findings. The present study addressed gaps that exist in the literature by assessing ALEs through a person-centered approach among a diverse sample of emerging adults. Given that the use of person-centered approaches is limited in the literature of ALEs and inconsistencies exist as to which ALEs tend to co-occur [45], replication of this study would be beneficial to better understand and confirm the classes of ALEs and their relationship to various types of mental health problems. It is important to consider that LCA identifies patterns within the specific sample and variables analyzed rather than fixed, universal groupings [4]; therefore, different classes may emerge depending on the sample used. Additionally, information about ALEs was collected through participants’ retrospective reports; thus, the reports of ALEs are limited to the participants’ ability to recall and report ALEs that they experienced. Indeed, research suggests that memory recall can impact the reporting of prior experienced adversity and that reports of childhood trauma can differ depending on whether individuals are asked prospectively or retrospectively [46,47]. Future research should aim to examine prospective reports of ALEs rather than relying solely on retrospective reports to determine if this significantly impacts findings. Further, most participants in this study identified as female, and the sample was limited to psychology students at a single university in Southern California. This may limit the generalizability of the findings, highlighting the need for future research to include more diverse samples of emerging adults from broader populations with balanced sex representation. The results of this study should be interpreted with caution as the *Witnessing Adversity* and *High Adversity* classes had a relatively small number of individuals (< 5% of the sample), which may contribute to instability in findings and limit the ability to draw strong conclusions regarding these classes [48]. This may be due to the use of a relatively low risk university student sample. Future research should confirm our findings in a sample of emerging adults with greater risk for ALEs exposure. Lastly, the present study used raw scores to assess mental health problems, focusing on symptom severity rather than clinical versus non-clinical thresholds. This limits the conclusions we can draw about whether each class is associated with clinically significant mental health problems. Future research could build on these findings by examining classes of ALEs in relation to clinical versus non-clinical mental health outcomes, particularly in samples likely to include individuals with more severe mental health problems. Despite these limitations, this study is among the first to examine patterns of co-occurring adversities across a wide range of ALEs and their associations with multiple mental health outcomes among emerging adults

## Implications for research and practice

The knowledge base on ALEs is quickly expanding, which has progressed from assessing the impacts of single ALEs, to employing cumulative risk approaches, to now accounting for different patterns of co-occurring ALEs [8,18]. Despite these advancements, further research is needed to understand how specific types of co-occurring ALEs relate to psychopathology. This study expands on previous research by implementing advanced methods to investigate the specific configuration of ALEs and their relationship to mental health problems among a sample of diverse emerging adults. Building upon prior research [21–23], our findings suggest that specific ALEs, such as child maltreatment and adult victimization, may put individuals at similar risk as to those who experience generally higher numbers of ALEs. This points to the potential detrimental effects of experiencing child and adult victimization, and thus may help inform targeted prevention/intervention efforts by identifying those who are at greatest risk of developing mental health problems as a result of adversity exposure.

Our findings also highlight the importance of assessing the specificity of the types of ALEs that are experienced by individuals. Future research should assess patterns of ALEs to determine if profiles of co-occurring adversity are consistent across samples, especially considering documented mixed findings regarding the co-occurrence of ALEs [45]. Furthermore, this study utilized dichotomous indicators of ALEs, but more information is needed about how severity impacts the configurations of ALEs. Thus, it would be beneficial for future research to assess configurations of ALEs, taking the severity of each specific adverse experience into account.

Lastly, our findings also suggest a need to understand the relative importance of ALEs, as we show that certain ALEs may put individuals at greater risk for developing mental health problems relative to others. Currently, screening tools that assess adverse life experiences do not utilize weights for different ALEs, but rather they ask whether individuals were exposed to certain adversities; thus, each ALE is considered to have the same detrimental effect, and focus on the accumulation of these adversities, with greater endorsed adversities suggesting greater risk [49]. However, specific ALEs, such as caregiver emotional hostility, have been shown to outweigh the accumulation of other contextual risk factors (e.g., parental divorce and death of a family member) [50]. In the present study, our findings suggest that child maltreatment and adult victimization may be highly important ALEs associated with worse mental health outcomes among emerging adults. This finding highlights that it might be beneficial to have specific types of adversities carry greater weight in adversity screening tools, as they could potentially lead to worse outcomes. Studies are increasingly using machine learning algorithms to examine the relative importance of specific adversities on mental health outcomes [19,51]. Future research could leverage machine learning algorithms to improve our understanding of the individual weight of ALEs in predicting risk for various mental health outcomes among emerging adults. Obtaining this information would help improve screening tools to identify individuals who may be most at risk and in need of targeted prevention efforts.

## Conclusions

The present study sought to address a knowledge gap in configurations of ALEs that are related to psychopathologies within a diverse sample of emerging adults. Our person-centered approach uncovered five-class of adversity configurations: (1) *Low Adversity*, (2) *Witnessing Adversity*, (3) *Experiencing Death*, (4) *Child Maltreatment and Adult Victimization*, and (5) *High Adversity.* Individuals in the *High Adversity* and *Child Maltreatment and Adult Victimization* classes had higher levels of psychopathology severity across outcomes relative to other classes. Further research is needed to compare the effects of individual, configural, and cumulative ALEs on mental health outcomes among emerging adults.
